# Supplementary material for: Increased S. epidermidis in the airway-gut microbiome of infants with bronchopulmonary dysplasia
Source: bioRxiv. 2026 Apr 4:2026.04.03.715941. Preprint. [Version 1] doi: 10.64898/2026.04.03.715941 (PMC13060358; doi:10.64898/2026.04.03.715941)
Supplement: Supplement 1 [file media-1.pdf]

Supplemental Files: Increased *S. epidermidis* in the airway-gut microbiome of infants with bronchopulmonary dysplasia

Zenna Solomon[1,*], Megan Eno[2,*], Sharon C. Thompson[2,3], Stephanie L. Ranger[1,4], Jenny C Jin[1,4], Melody Zeng[1,4], Divya Keerthy[5], Stefan Worgall[1,4,6], Elizabeth L. Johnson[2,3], Andrea Heras[1,4]

[1]Department of Pediatrics, Weill Cornell Medicine, New York, NY
[2]Division of Nutritional Sciences, Cornell University, Ithaca, NY
[3]Howard Hughes Medical Institute, Cornell University, Ithaca, NY
[4]Drukier Institute for Children's Health, Weill Cornell Medicine, New York, NY
[5]Neonatal and Perinatal Medicine, NewYork-Presbyterian Queens, Flushing, NY
[6]Department of Genetic Medicine, Weill Cornell Medicine, New York, NY

Supplemental Figures 1 – 4
Supplemental Tables 1 – 2

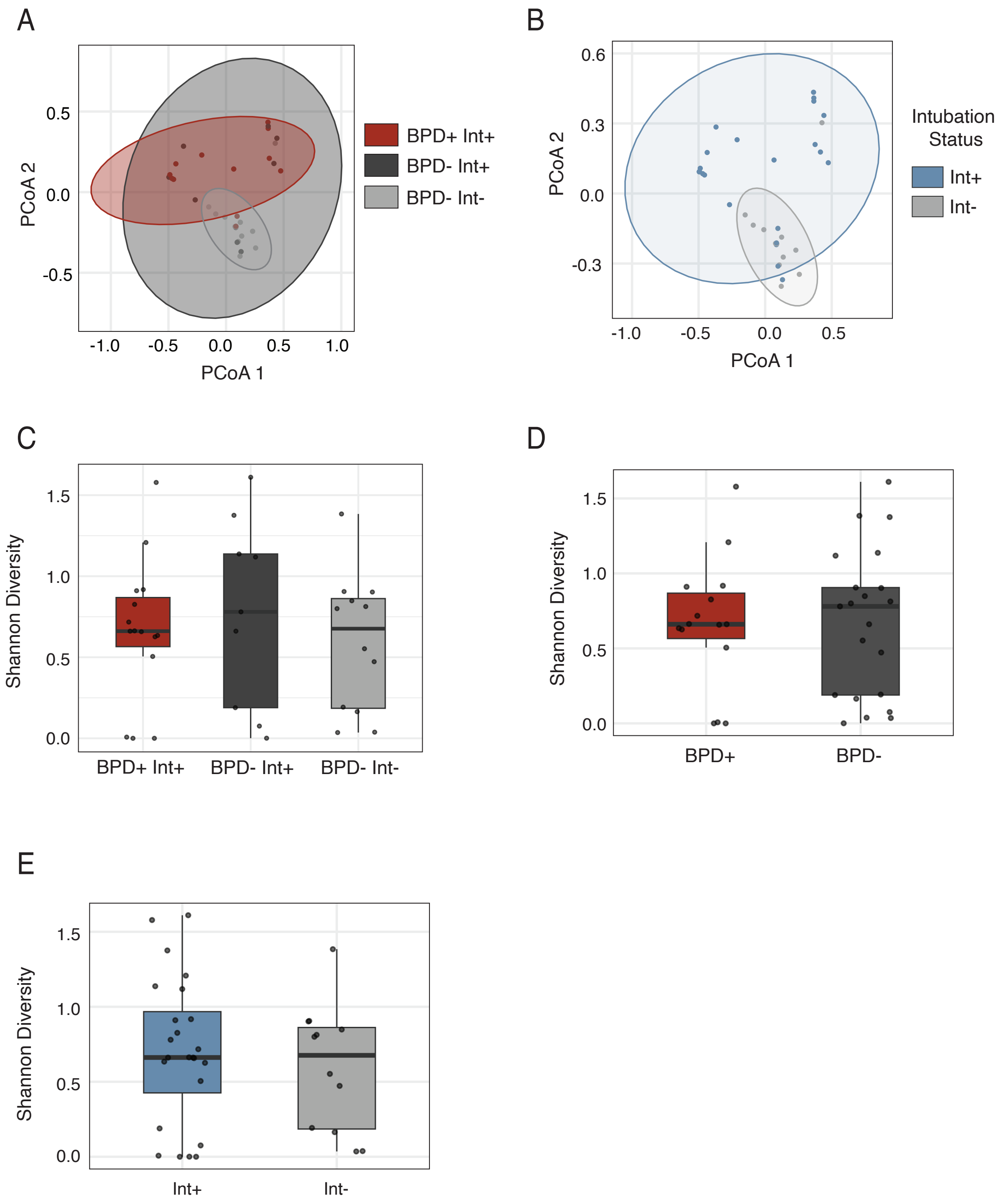

Supplemental Figure 1 – Alpha and beta diversity analysis by BPD and intubation status.

(A) Bray-Curtis dissimilarity among BPD$^+$ Int$^+$, BPD$^-$ Int$^+$, and BPD$^-$ Int$^-$ infants ($R^2 = 0.094$, F = 1.72, p = 0.022), (B) Int$^+$ and Int$^-$ infants (PERMANOVA: $R^2 = 0.072$, F. = 2.636, p = 0.007) using the first sample per infant. (C) Shannon diversity among BPD$^+$ Int$^+$, BPD$^-$ Int$^+$, and BPD$^-$ Int$^-$ infants (Kruskal-Wallis: $\chi^2 = 0.625$, df = 2, p = 0.73), (D) BPD$^+$ and BPD$^-$ infants (Wilcoxon rank-sum: W = 148, p = 0.773), and (E) Int$^+$ and Int$^-$ infants (Wilcoxon rank-sum: W = 156, p = 0.6995) using the first sample per infant.

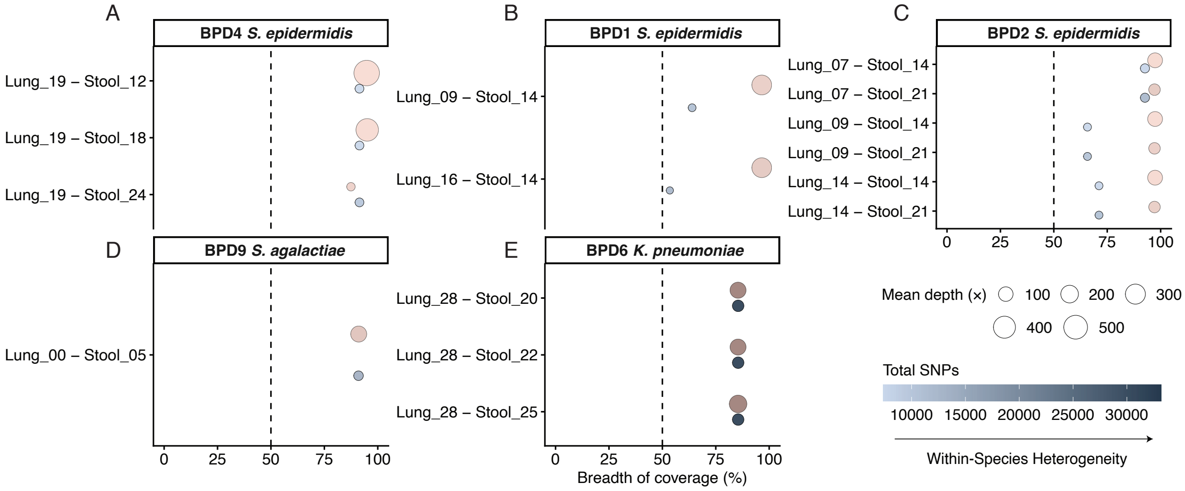

Supplemental Figure 2 – Species sharing analysis using minimap2 between lung and stool metagenomes. (A) Infant – BPD4, (B) Infant – BPD1, (C) Infant – BPD2, (D) Infant – BPD9, and (E) Infant – BPD6 at different days of life. On the x-axis is the breadth of coverage between the lung and stool metagenomes and the given species reference genome. Also represented on the x-axis is a dotted line denoting a threshold of 50% breadth of coverage. Color of the point represents the site (red = stool, blue = lung), and size of the point represents the depth of coverage of the alignment between lung, stool, and the given species genome.

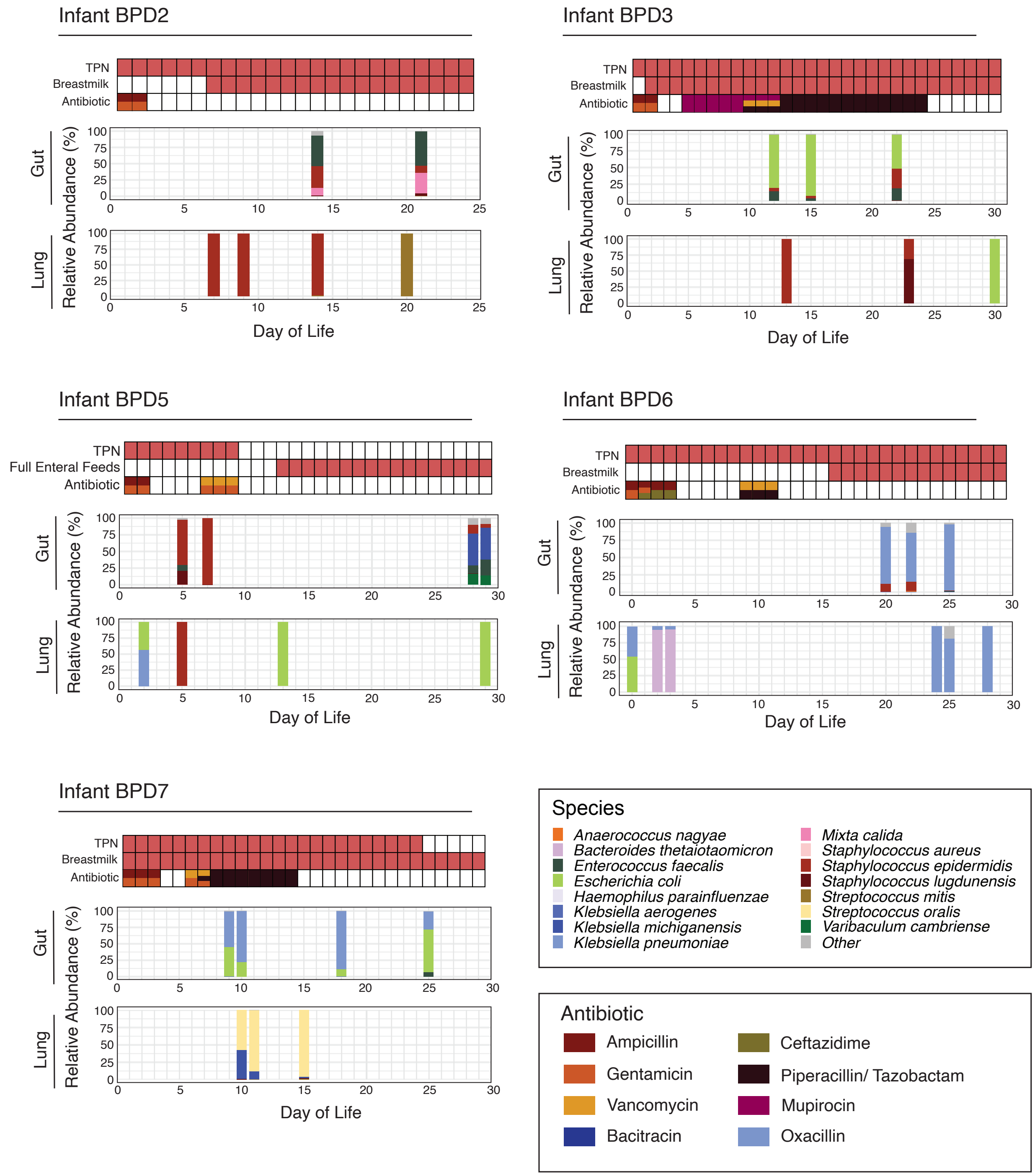

Supplemental Figure 3 – Microbial community shifts in the gut and lower airway microbiome of individual infants over periods of feeding changes and antibiotic intake. The top 15 species within the samples plotted are denoted in the legend with the other species combined into the “Other” category. If two samples were collected for one infant on the same day, the first sample was plotted.

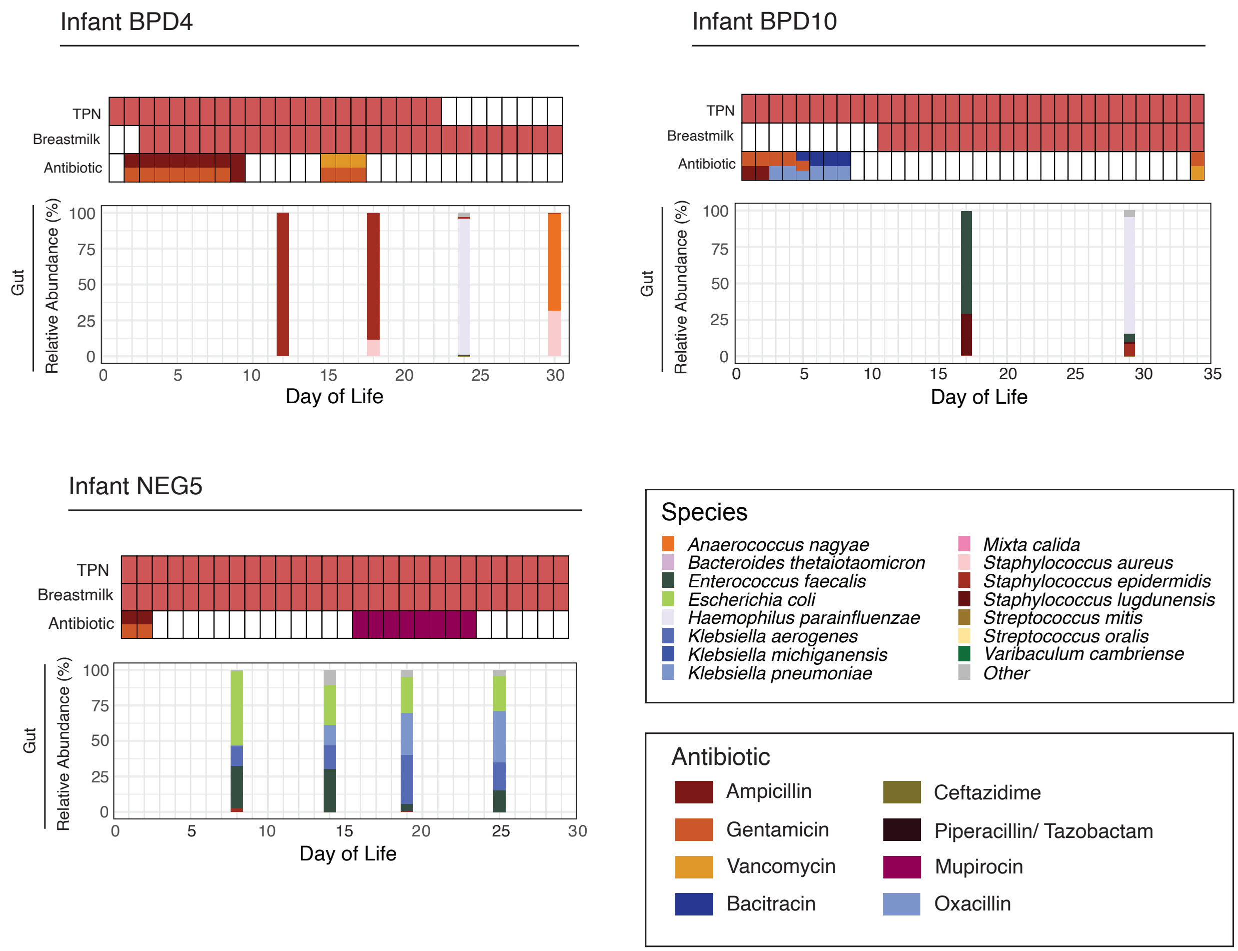

Supplemental Figure 4– Microbial community shifts in the gut microbiome of individual infants over periods of feeding changes and antibiotic intake. The top 15 species within the samples plotted are denoted in the legend with the other species combined into the "Other" category. If two samples were collected for one infant on the same day, the first sample was plotted.

Supplemental Table 1 – Species tracking alignments and their NCBI RefSeq IDs.

query1	query2	lineage	NCBI RefSeq
lung_BPD9_0	stool_BPD9_5	*Streptococcus agalactiae*	GCF_001552035.1
lung_BPD2_7	stool_BPD2_21	*Staphylococcus epidermidis*	GCF_006094375.1
lung_BPD2_9	stool_BPD2_21	*Staphylococcus epidermidis*	GCF_006094375.1
lung_BPD6_0	stool_BPD6_22	*Klebsiella pneumoniae*	GCF_000240185.1
lung_BPD6_28	stool_BPD6_22	*Klebsiella pneumoniae*	GCF_000240185.1
lung_BPD6_28	stool_BPD6_25	*Klebsiella pneumoniae*	GCF_000240185.1
lung_BPD6_28	stool_BPD6_25_timepoint_2	*Klebsiella pneumoniae*	GCF_000240185.1
lung_BPD2_9	stool_BPD2_14	*Staphylococcus epidermidis*	GCF_006094375.1
lung_BPD2_7	stool_BPD2_14	*Staphylococcus epidermidis*	GCF_006094375.1
lung_BPD2_14	stool_BPD2_21	*Staphylococcus epidermidis*	GCF_006094375.1
lung_BPD6_0	stool_BPD6_25_timepoint_2	*Klebsiella pneumoniae*	GCF_000240185.1
lung_BPD6_0	stool_BPD6_25	*Klebsiella pneumoniae*	GCF_000240185.1
lung_BPD4_19	stool_BPD4_12	*Staphylococcus epidermidis*	GCF_006094375.1
lung_BPD5_5	stool_BPD5_28	*Staphylococcus epidermidis*	GCF_006094375.1
lung_BPD4_19	stool_BPD4_24	*Staphylococcus epidermidis*	GCF_006094375.1
lung_BPD5_5	stool_BPD5_29	*Staphylococcus epidermidis*	GCF_006094375.1
lung_BPD2_14	stool_BPD2_14	*Staphylococcus epidermidis*	GCF_006094375.1
lung_BPD6_28_timepoint_2	stool_BPD6_22	*Klebsiella pneumoniae*	GCF_000240185.1
lung_BPD3_13	stool_BPD3_15	*Staphylococcus epidermidis*	GCF_006094375.1
lung_BPD6_28_timepoint_2	stool_BPD6_25	*Klebsiella pneumoniae*	GCF_000240185.1
lung_BPD6_28	stool_BPD6_20	*Klebsiella pneumoniae*	GCF_000240185.1
lung_BPD3_30	stool_BPD3_15	*Escherichia coli*	GCF_000005845.2
lung_BPD6_28_timepoint_2	stool_BPD6_25_timepoint_2	*Klebsiella pneumoniae*	GCF_000240185.1
lung_BPD3_30	stool_BPD3_15_timepoint_2	*Escherichia coli*	GCF_000005845.2
lung_BPD6_2	stool_BPD6_25	*Klebsiella pneumoniae*	GCF_000240185.1
lung_BPD6_2	stool_BPD6_25_timepoint_2	*Klebsiella pneumoniae*	GCF_000240185.1
lung_BPD5_5	stool_BPD5_7	*Staphylococcus epidermidis*	GCF_006094375.1
lung_BPD6_28_timepoint_2	stool_BPD6_20	*Klebsiella pneumoniae*	GCF_000240185.1
lung_BPD4_19	stool_BPD4_18	*Staphylococcus epidermidis*	GCF_006094375.1
lung_BPD5_29	stool_BPD5_29	*Escherichia coli*	GCF_000005845.2
lung_BPD6_0	stool_BPD6_20	*Klebsiella pneumoniae*	GCF_000240185.1
lung_BPD6_2	stool_BPD6_22	*Klebsiella pneumoniae*	GCF_000240185.1
lung_BPD3_13	stool_BPD3_15_timepoint_2	*Staphylococcus epidermidis*	GCF_006094375.1
lung_BPD6_3	stool_BPD6_25	*Klebsiella pneumoniae*	GCF_000240185.1
lung_BPD6_3	stool_BPD6_25_timepoint_2	*Klebsiella pneumoniae*	GCF_000240185.1
lung_BPD6_24	stool_BPD6_25	*Klebsiella pneumoniae*	GCF_000240185.1
lung_BPD3_23	stool_BPD3_15	*Staphylococcus epidermidis*	GCF_006094375.1
lung_BPD4_19_timepoint_2	stool_BPD4_18	*Staphylococcus aureus*	GCF_000013425.1

lung_BPD6_3	stool_BPD6_22	*Klebsiella pneumoniae*	GCF_000240185.1
lung_BPD3_30	stool_BPD3_22	*Escherichia coli*	GCF_000005845.2
lung_BPD6_24	stool_BPD6_22	*Klebsiella pneumoniae*	GCF_000240185.1
lung_BPD6_24	stool_BPD6_25_timepoint_2	*Klebsiella pneumoniae*	GCF_000240185.1
lung_BPD6_2	stool_BPD6_20	*Klebsiella pneumoniae*	GCF_000240185.1
lung_BPD7_11	stool_BPD7_25	*Klebsiella michiganensis*	GCF_015139575.1
lung_BPD6_25_timepoint_2	stool_BPD6_25	*Klebsiella pneumoniae*	GCF_000240185.1
lung_BPD3_30	stool_BPD3_12	*Escherichia coli*	GCF_000005845.2
lung_BPD5_29	stool_BPD5_28	*Escherichia coli*	GCF_000005845.2
lung_BPD7_10	stool_BPD7_25	*Klebsiella michiganensis*	GCF_015139575.1
lung_BPD3_23	stool_BPD3_15_timepoint_2	*Staphylococcus epidermidis*	GCF_006094375.1
lung_BPD6_25_timepoint_2	stool_BPD6_22	*Klebsiella pneumoniae*	GCF_000240185.1
lung_BPD7_1	stool_BPD7_25	*Klebsiella pneumoniae*	GCF_000240185.1
lung_BPD6_25_timepoint_2	stool_BPD6_25_timepoint_2	*Klebsiella pneumoniae*	GCF_000240185.1
lung_BPD1_9	stool_BPD1_14	*Staphylococcus epidermidis*	GCF_006094375.1
lung_BPD6_3	stool_BPD6_20	*Klebsiella pneumoniae*	GCF_000240185.1
lung_BPD7_4	stool_BPD7_25	*Klebsiella pneumoniae*	GCF_000240185.1
lung_BPD4_19_timepoint_2	stool_BPD4_30	*Staphylococcus aureus*	GCF_000013425.1
lung_BPD6_25	stool_BPD6_25	*Klebsiella pneumoniae*	GCF_000240185.1
lung_BPD6_25	stool_BPD6_22	*Klebsiella pneumoniae*	GCF_000240185.1
lung_BPD6_25_timepoint_2	stool_BPD6_20	*Klebsiella pneumoniae*	GCF_000240185.1
lung_BPD1_16	stool_BPD1_14	*Staphylococcus epidermidis*	GCF_006094375.1

Supplemental Table 2 – *Staphyloccocus epidermidis* strains utilized in strain tracking analysis.

Strain	NCBI RefSeq
Staphyloccocus epidermidis ATCC 14990	GCF_006094375.1
Staphyloccocus epidermidis B1276912	GCF_019329745.1
Staphyloccocus epidermidis B1272014	GCF_019329665.1
Staphyloccocus epidermidis UMCG345	GCF_045940975.1
Staphyloccocus epidermidis UMCG350	GCF_045939605.1
Staphyloccocus epidermidis B1230143	GCF_019329425.1
Staphyloccocus epidermidis AR4072	GCF_031326165.1
Staphyloccocus epidermidis IRL01	GCF_009685135.1
Staphyloccocus epidermidis B1266875	GCF_019329585.1
Staphyloccocus epidermidis TMDU-265	GCF_024204785.1
Staphylococcus hominis FDAARGOS_575	GCF_003812505.1
Staphylococcus capitis DSM 6717	GCF_040739365.1
Staphylococcus epidermidis AU12-03	NA
